# Validation of a comprehensive long-read sequencing platform for broad clinical genetic diagnosis

**DOI:** 10.3389/fgene.2025.1499456

**Published:** 2025-05-02

**Authors:** Siddhartha Sen, Hillary P. Handler, Alec Victorsen, Zach Flaten, Aidan Ellison, Todd P. Knutson, Sarah A. Munro, Ryan J. Martinez, Charles John Billington, Jennifer J. Laffin, Sarah Bray, Pawel Mroz, Sophia Yohe, Andrew C. Nelson, Matthew Bower, Bharat Thyagarajan

**Affiliations:** ^1^ University of Minnesota Health Sciences, University of Minnesota Medical Center, Minneapolis, MN, United States; ^2^ Molecular Diagnostics Laboratory, Fairview Health, University of Minnesota Medical Center, Minneapolis, MN, United States; ^3^ Minnesota Supercomputing Institute, University of Minnesota, Minneapolis, MN, United States

**Keywords:** long-read sequencing, Oxford Nanopore Technologies, clinical genomics, whole genome sequencing, Tandem repeat expansions, complex structural variants

## Abstract

Though short read high-throughput sequencing, commonly known as Next-Generation Sequencing (NGS), has revolutionized genomics and genetic testing, there is no single genetic test that can accurately detect single nucleotide variants (SNVs), small insertions/deletions (indels), complex structural variants (SVs), repetitive genomic alterations, and variants in genes with highly homologous pseudogenes. The implementation of a unified comprehensive technique that can simultaneously detect a broad spectrum of genetic variation would substantially increase efficiency of the diagnostic process. The current study evaluated the clinical utility of long-read sequencing as a comprehensive genetic test for diagnosis of inherited conditions. Using Oxford Nanopore Technologies long read nanopore sequencing, we successfully developed and validated a clinically deployable integrated bioinformatics pipeline that utilizes a combination of eight publicly available variant callers. A concordance assessment comparing the known variant calls from a well-characterized, benchmarked sample called NA12878 from the National Institute of Standards and Technology (NIST) with the variants detected by our pipeline for this sample, determined that the analytical sensitivity of our pipeline was 98.87% and the analytical specificity exceeded 99.99%. We then evaluated our pipeline’s ability to detect 167 clinically relevant variants from 72 clinical samples. This set of variants consisted of 80 SNVs, 26 indels, 32 SVs, and 29 repeat expansions, including 14 variants in genes with highly homologous pseudogenes. The overall detection concordance for these clinically relevant variants was 99.4% (95% CI: 99.7%–99.9%). Importantly, in addition to detecting known clinically relevant variants, in four cases, our pipeline yielded valuable additional information in support of clinical diagnoses that could not have been established using short-read NGS alone. Our findings suggest that long-read sequencing is successful in identifying diverse genomic alterations and that our pipeline functions well as the basis for a single diagnostic test for patients with suspected genetic disease.

## Introduction

Massively parallel NGS techniques have revolutionized the molecular diagnosis of rare genetic conditions (Fernandez-Marmiesse et al., 2018) (PMID: 28721829). Specifically, hybridization-based target enrichment in conjunction with short-read sequencing has emerged as a leading molecular diagnostic technique in clinical genetic laboratories (Singh, 2022) (PMID: 35885445). However, the length of short reads creates several limitations that include mapping ambiguity of highly repetitive and/or ‘GC’ rich genome regions as well as limited the ability to accurately sequence large complex SVs (Zavodna et al., 2014) (PMID: 25436869). Patients with rare disorders caused by complex variants typically undergo multiple discrete rounds of genetic testing. This can lead to delays in diagnosis and a significant financial burden for patients. Long-read sequencing methodology capable of sequencing DNA fragments that are tens of thousands of nucleotides in length is an attractive technology that can address the limitations of short-read technology and was named technology of the year in 2022 (Marx, 2023) (PMID: 36635542). Two companies, Oxford Nanopore Technologies and Pacific Biosystems (PacBio), have pioneered the commercialization of long-read technologies. When first introduced, nanopore long-read sequencing showed higher error rates, excluding it as a candidate technology capable of replacing short read-based sequencing for clinical diagnostics (Lu et al., 2016) (PMID: 27646134). However, with recent advances in chemistry, flow cell technology (R10), and base-calling algorithms, modal read accuracy has greatly improved and SNV F1 scores are over 98% (Ni et al., 2023; Liu et al., 2021; Srivathsan et al., 2024) (PMID: 37025654, 33612390, 38041646).

While there are several reports demonstrating the advantages of long-read sequencing technologies for detection of CNVs, SVs, and variants in genomic regions that have historically been difficult to sequence using short-read technologies, adoption of long-read sequencing into clinical practice has been limited (Zavodna et al., 2014) (PMID: 25436869). In a pilot study, researchers successfully used a nanopore-based workflow to perform ultra-rapid whole genome sequencing on critically ill patients, resulting in diagnoses of rare genetic diseases in approximately 8 hours (Gorzynski et al., 2022) (PMID: 35020984). In another recent study, a pathogenic SV was identified in a patient with multiple neoplasia and cardiac myxomata using long-read sequencing, in whom previous targeted short-read sequencing was negative, thereby resulting in a clinical diagnosis (Merker et al., 2018) (PMID: 28640241). Another pilot study demonstrated the usefulness of ONT-based targeted long-read sequencing for the molecular testing and diagnosis of short tandem repeat (STR) expansion disorders (Stevanovski et al., 2022) (PMID: 35245110). Thus, ONT has now become a high-throughput, high-fidelity long-read sequencing technology which can be used for rapid diagnosis in a clinical setting. While the high cost of long-read sequencing has been a barrier to clinical implementation, the costs of these technologies (both ONT and PacBio) have been decreasing (Hook and Timp, 2023) (PMID: 37161088). Another major hurdle to the wide-spread adoption of long-read sequencing into clinical testing is the lack of integrated workflows allowing comprehensive detection of different types of genetic variants, which is essential for establishing a robust clinical assay for inherited disorders. There are currently numerous bioinformatics tools that are specifically employed to identify particular types of genetic variants (e.g., SNVs, CNVs, repeat expansions, etc.) using Nanopore long reads.

We previously developed and successfully integrated a short-read sequencing and a copy number variation (CNV) detection pipeline into a broad-based NGS platform for clinical testing to meet the genetic testing needs in the Molecular Diagnostics Laboratory (MDL) at the University of Minnesota (UMN) (Onsongo et al., 2016; Hartman et al., 2019) (PMID: 27597741, 30891420). A current unmet need shared by MDL and other institutions is the implementation of a single comprehensive genetic test that can accurately detect SNVs, small indels, complex SVs, repetitive genomic alterations, and variants in genes with highly homologous pseudogenes. Neurology is one clinical area where this need is evident, particularly with respect to the diagnosis of hereditary cerebellar ataxias. Patients with ataxia often incur multiple rounds of genetic testing and significant financial burden before receiving a diagnosis, if they receive a clear diagnosis at all. Given the relatively low diagnostic rates and long diagnostic odysseys experienced by this patient population, a recent review proposed that the theoretical best approach to genetic diagnosis of hereditary cerebellar ataxia would be to implement a long-read sequencing platform that could supersede a sequential testing approach altogether in all cases where a monogenic cause is not highly suspected (Rudaks et al., 2024) (PMID: 38760634). The goal of this study is to address this unmet need for patients with diverse genetic conditions caused by a wide range of genetic mutations. Here, we describe the development of a comprehensive genetic testing platform that leverages the advantages of long-read sequencing and uses several variant callers for a complete analysis of the full spectrum of disease-causing genomic alterations for clinical diagnostics.

## Methods

### Concordance studies

A benchmarked sample, NA12878/HG001 (v4.2.1), was purchased from NIST (https://www.nist.gov/programs-projects/genome-bottle). The NA12878 sample represents a single female genome that has been extensively characterized using multiple genomic analysis platforms (Zook et al., 2016) (PMID: 27271295). Using the custom workflow described below, NA12878 was prepared and sequenced on an Oxford Nanopore Technologies PromethION-24 at the UMN Advanced Research and Diagnostics Laboratory (ARDL). A concordance analysis was performed to determine the ability of our ONT sequencing pipeline to detect the well-characterized SNV and indel variants present in the NA12878 sample. This concordance analysis was restricted to exonic variants (n = 26,584) in annotated human genes associated with clinical phenotypes. This restriction was performed by intersecting the NA12878 VCF file with a BED file (DOI: 10.5281/zenodo.14532101) containing exonic coordinates for 5631 clinically relevant genes (Supplementary Table S1) using BEDTools (Quinlan and Hall, 2010) (PMID: 20110278). This list of 5631 clinically relevant genes is regularly updated and it is curated using data from publicly available databases such as OMIM as well as new publications in the relevant medical literature identified via periodic targeted PubMed searches. Concordance analysis was performed by comparing VCF files and identifying exact matches at the chromosome (CHROM), position (POS), reference (REF), and alternate (ALT) columns using R (v4.0.4).

### Clinical sample selection and storage

Seventy-two clinical samples previously analyzed at the UMN MDL were selected for nanopore sequencing (Supplementary Table S2). These samples were chosen as a representation of diverse genetic variation. Each sample had at least one clinically relevant abnormal repeat expansion, SNV, indel, SV, or variant in a gene with a highly homologous pseudogene. The available specimens were either extracted DNA or buffy coat stored at −80°C.

### Sample preparation

For 56 samples, DNA was purified from buffy coats on an Autogen Flexstar; 16 samples had been previously extracted [Qiagen DNeasy Blood & Tissue Kit (Cat No. 69506)]. Extracted DNA was concentrated using an Eppendorf Vacufuge plus at room temperature. In most cases, 4 µg of DNA was diluted into 150 µL water and sheared by centrifugation in Covaris g-TUBEs for 30 s at 1,250 g. Sheared DNA was characterized using an Invitrogen Qubit using the 1x dsDNA BR assay, and on an Agilent Tapestation. Ideally, samples had approximately 80% of the sheared fragments between 8 kb and 48.5 kb in length. Though Tapestation fragment size did not directly correlate with N50, the Tapestation fragment size was used as a quality check to estimate optimal levels of DNA shearing prior to sequencing. Samples were prepared for sequencing using the Oxford Nanopore Ligation Sequencing kit V14 using 3 µg of sheared DNA.

### Whole genome sequencing

Samples were sequenced on a PromethION-24 and run on a single flow cell (R10.4.1 with the E8.2 motor protein) for approximately 5 days, with daily washing and reloading. For each sample, one library was built and split into four to five ∼300 ng aliquots. One flow cell was used to sequence four library aliquots. Each aliquot was sequenced overnight. The following day, the flow cell was washed for at least an hour before a new aliquot was added. Each flow cell was run for a total of 100 h. No adaptive sequencing was used. Several versions of MinKNOW were used depending on when the samples were sequenced. The versions used were 22.12.5, 23.04.6, 23.07.8, 23.07.12, 23.11.4, and 23.11.7.

### Bioinformatics analysis

Data was processed at the Minnesota Supercomputing Institute. Default parameters were used for all software unless otherwise noted. POD5 files were processed using Dorado(v0.3.4+5f5cd02+cu118) with a minimum q-score of 9. The Dorado basecalling model used was dna_r10.4.1_e8.2_400bps_sup@v4.2.0. Methylation analysis was not performed in this study. Unaligned BAMs were aligned to the hg19 genome using Nanopore’s epi2me wf-alignment workflow(v0.3.3). Sequencing depth was calculated using Mosdepth(v0.3.3) (Pedersen and Quinlan, 2018) (PMID: 29096012) and aligned N50s were calculated using Cramino(v0.9.9) (De Coster and Rademakers, 2023) (PMID: 37171891). Read quality was assessed with NanoPlot(v1.44.0) (De Coster and Rademakers, 2023) (PMID: 37171891).

### Variant calling pipeline

SNVs were identified with Clair3 as the default genotyper using the epi2me wf-human-variation workflow (v1.2.0) (Zheng et al., 2022) (PMID: 38177392). Insertions/deletions/inversions (INS/DEL/INV) were identified using NanoVar(v1.5.1) (Tham et al., 2020) (PMID: 32127024) and DeBreak (v1.0.2) (Chen Y. et al., 2023) (PMID: 36650186). SVs identified by DeBreak were limited to a minimum of 100 bp in length while NanoVar was used to identify SVs > 50 bp. INS/DEL < 50 bp were identified using the Clair3 genotyper. CNVs on the sex chromosomes were identified using CNVpytor(1.3.1) (Suvakov et al., 2021) (PMID: 34817058) with 1 kbp bins, which were also filtered by: Q0 < 0.5, p_N < 0.5, and p-values < 0.001; choosing these parameters allowed for the highest quality calls for review. Autosomal CNVs were called by QDNAseq (1 kbp bin size), which is included in the epi2me workflow (v1.10.1).

**FIGURE 1:**
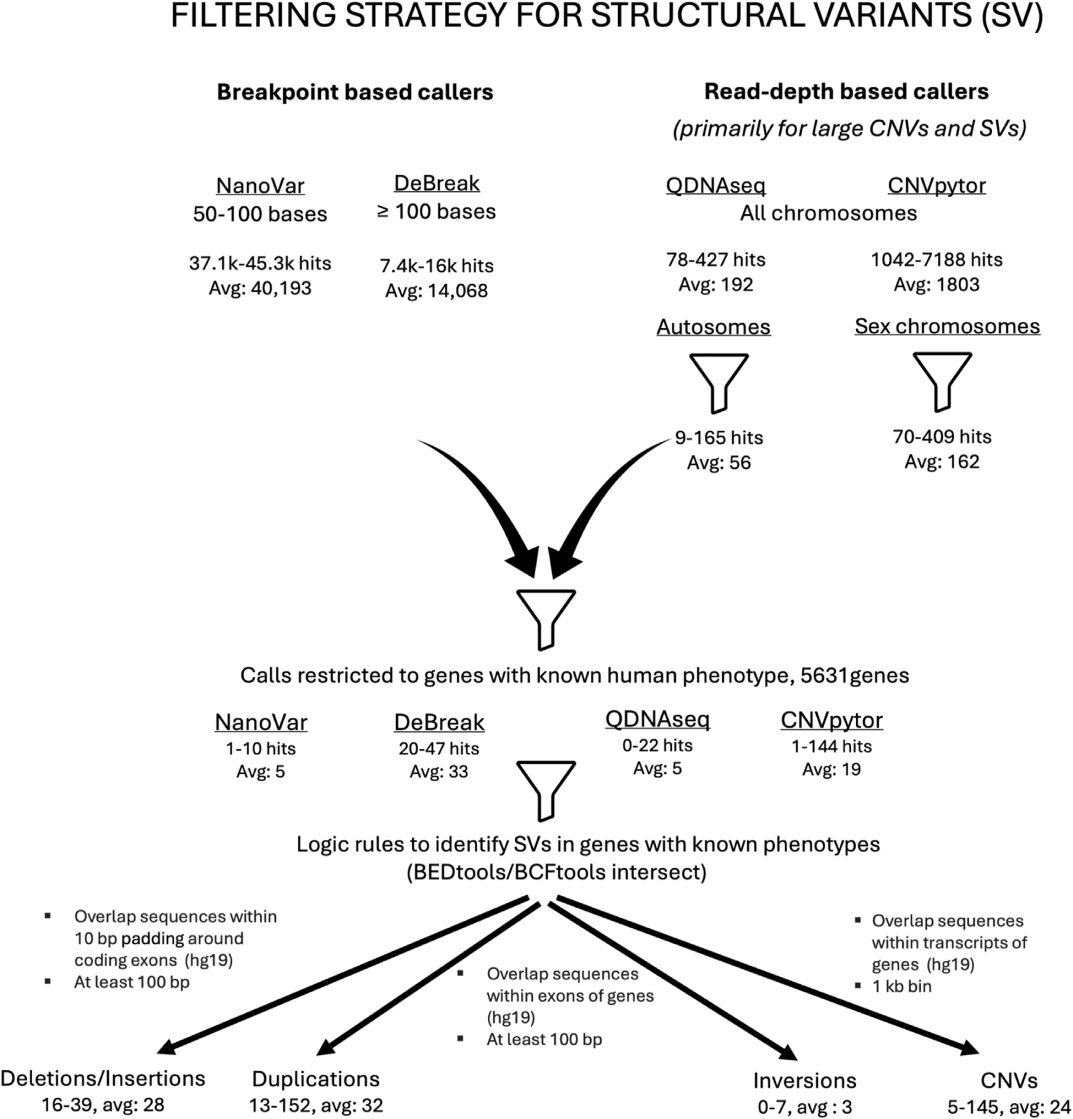
Filtering strategy employed in the detection of clinically relevant structural variants. Flow chart outlining the ONT pipeline’s SV call filtering strategy. A combination of breakpoint-based callers (NanoVar and DeBreak) as well as read-depth callers (QDNAseq and CNVpytor) was used to identify structural variants (SVs). Before any filters were applied, there was an average of approximately 40,000 hits for small SVs detected by NanoVar and approximately 14,000 by DeBreak. For the large SVs and CNVs, there was an average of 192 hits identified by QDNAseq and 1803 by CNVpytor, respectively. When filtered for autosomes and sex chromosomes, the average numbers of large SVs/CNVs were 56 for autosomes and 162 for sex chromosomes, respectively. The next filtering step involved restricting the SV calls to genes that have a human phenotype, which again diminished the total number of SVs. The last filtering step involved application of a set of logic rules resulting in a further reduction in the number of SVs. The SVs that remained at the end of all the filtering steps were subjected to a final review and classified.

A structured filtering approach was developed to accurately identify clinically relevant SVs (Figure 1). In the first filtering step, each of the caller’s outputs were restricted to a subset of calls related to its variant calling strengths. The next filtering step involved narrowing the list of calls to variant types that could mechanistically cause Mendelian disease. For example, an inversion impacting a subset of coding exons within a gene would advance to the next filtering step, but a complete gene inversion with intergenic breakpoints would be excluded. Another filtering step was then performed to restrict the calls to genes that have a human phenotype. This list of 5,631 genes associated with clinically relevant human phenotypes is regularly updated and it is curated using data from publicly available databases such as OMIM as well as new publications in the relevant medical literature identified via periodic targeted PubMed searches (Supplementary Table S1). A final set of logic rules was applied using a BEDtools/BCFtools intersect (Quinlan and Hall, 2010; Danecek et al., 2021) (PMID: 20112078, 33590861) as the last step to accurately identify and detect a pathogenic SV.

**FIGURE 2:**
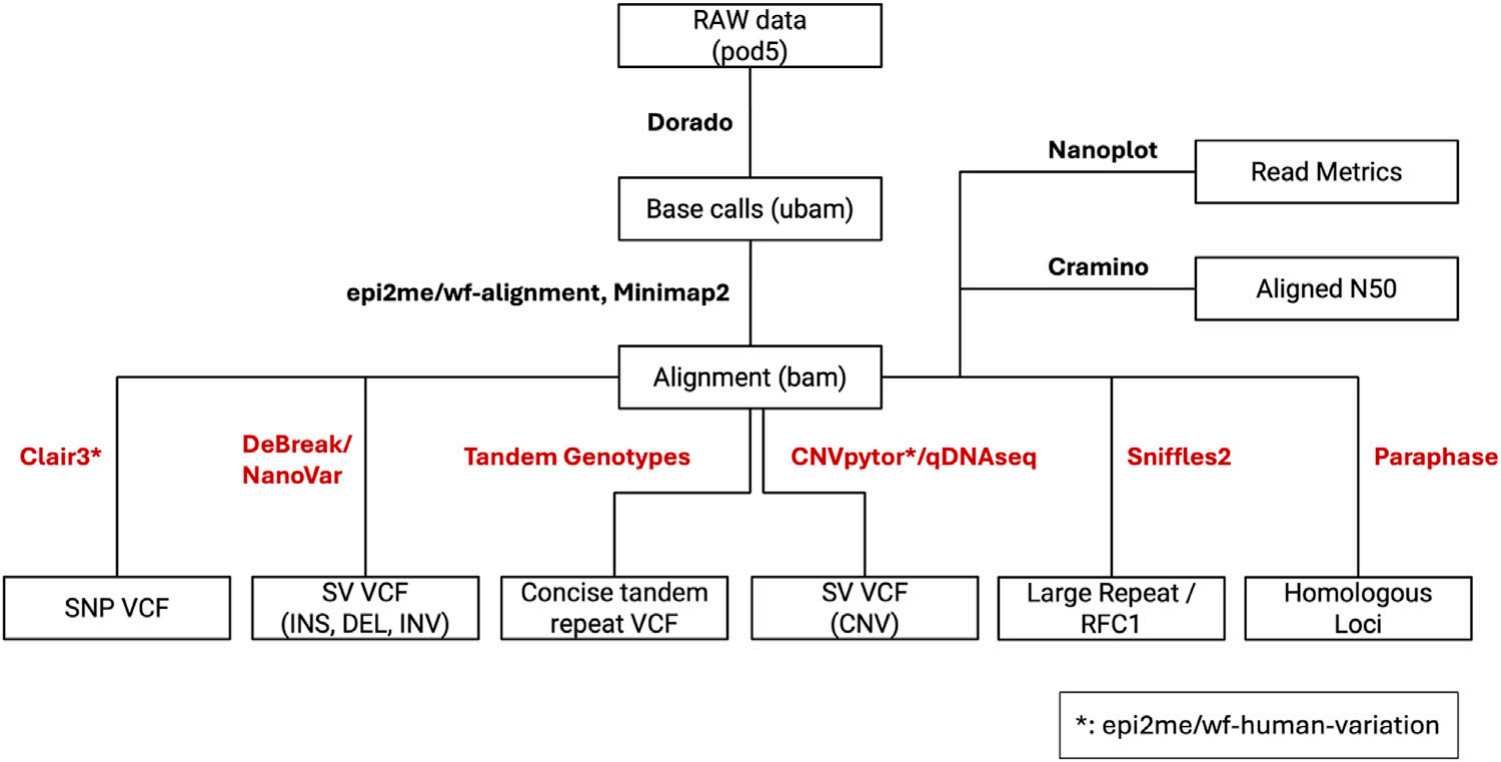
Custom nanopore variant calling pipeline. The boxes depict file names and file types. The connecting lines between boxes are labeled with the pipeline components that require and generate respective input and output file types. The pipeline outputs multiple unique variant call files (VCFs) that will be combined for clinical analysis.

Samples were processed with Paraphase (v3.1.1) (Chen X. et al., 2023) (PMID: 36669496) for assessment of variants in genes like *PMS2* and *STRC*, that are known to have homologous pseudogenes. Tandem repeat expansions were called using Tandem Genotypes (v1.9.1) (Mitsuhashi et al., 2019) (PMID: 30890163) and Sniffles2 (v2.0.7) (Sedlazeck et al., 2018) (PMID: 29713083). For Tandem Genotypes, reads surrounding loci of interest were realigned using LAST (v1256) (Kielbasa et al., 2011) (PMID: 21209072). A schematic representation of the pipeline is shown in Figure 2.

All clinical analyses and reporting infrastructure in our laboratory currently use hg19 as human reference. In order to maintain consistency and ensure that results can be accurately compared to our current clinical pipelines, sequencing data generated by our ONT-based pipeline was also analyzed using hg19 as the reference genome.

## Results

### NA12878 concordance

Since SNVs and small indels are the most common clinically relevant genomic alterations, we compared how the ONT-based pipeline performed when compared to our current short-read based clinical pipeline. Our ONT-based long-read pipeline was able to correctly identify 26,210 of the 26,584 gold-standard NA12878 variants across exons of known annotated coding genes, which corresponds to an analytical sensitivity of 98.87% and an analytical specificity exceeding 99.99%. In comparison, when our current clinically validated short-read pipeline was compared with the NA12878 sample, it showed a comparable analytical sensitivity of 99.59% and a specificity exceeding 99.99% on the same set of variants (Table 1).

**TABLE 1:** Clinically significant variant concordance by variant type.

Variant type	Concordance	CI (95%)
SNV	1.0000	0.95–1.0
Indel	0.9615	0.811–0.9932
SV	1.0000	0.8928–1.0
Repeat	1.0000	0.883–1.0
Overall	0.9940	0.9669–0.9989

To assess and compare the analytical sensitivity and specificity for detection of SNVs and indels, the set of 26,584 gold-standard NA12878 variants was analyzed separately based on variant type. For the 25,514 SNVs in this dataset, the analytical sensitivity was 98.91% and the analytical specificity exceeded 99.99%. For the 1,070 indel calls in this dataset, the analytical sensitivity is 97.81% and the analytical specificity exceeds 99.99% (Table 1).

While the National Institute of Standards and Technology (NIST) does have available samples with well-characterized SVs, these samples were not used to assess the analytical detection abilities of our pipeline. The available samples from healthy individuals do not have enough CNV or SV calls in coding regions of clinically relevant genes to accurately evaluate the analytical sensitivity and specificity of our pipeline.

### Evaluation of variant detection tools

Prior to testing our pipeline’s ability to detect a diverse spectrum of clinically relevant genetic variation, we assessed the functionality of several variant detection tools on a pilot set of clinical samples. Clair3 accurately identified all SNVs and small indels in the pilot dataset. Therefore, Clair3 was included in our pipeline as the default genotyper for small genetic alterations.

With respect to detecting SVs, we systematically evaluated the functionality of several available breakpoint callers (Supplementary Table S3). In our initial assessment of SV breakpoint callers, we evaluated eight clinical samples with known complex SVs, including a combination of deletions, duplications, inversions, and/or translocations. Across the eight samples with complex SVs, there were 29 known breakpoints. Of the tools evaluated, NanoVar was the most sensitive tool for breakpoint identification, with a detection rate of 93.1%. While DeBreak’s SV detection rate (86.2%) was lower than NanoVar’s, DeBreak was superior in characterizing the type of rearrangement occurring at a particular breakpoint rather than assigning it a generic classification such as “breakpoint not determined.” In contrast, SVIM and Sniffles2 displayed significantly inferior SV detection performance, with SV breakpoint detection rates of 58.6% and 6.9%, respectively. Therefore, based on the performance of the callers tested within our clinical sequencing environment (non-targeted capture with a target minimum genome-wide coverage of 30x), DeBreak was selected as the pipeline’s primary SV caller (for SVs >100 bases), with Nanovar implemented specifically to identify SV calls between 50-100 bases in size.

For detection of primarily larger SVs and CNVs that are not mediated by detectible breakpoints, we assessed two read-depth based callers, QDNAseq and CNVpytor. We found that QDNAseq performed best for autosomes, while CNVpytor performed best for sex chromosomes.

As clinically significant repeat expansion variants are a unique subset of SV that are notoriously difficult to accurately characterize by short-read NGS, we also assessed the functionality of a tool specifically designed to detect and quantify repeat expansion content. The Tandem Genotypes tool performed well for all loci assessed except for the pentanucleotide repeat expansions in *RFC1* that cause cerebellar ataxia, neuropathy, vestibular areflexia syndrome (CANVAS). In search of a bioinformatics tool capable of identifying clinically relevant variants specifically in *RFC1*, we begin by reevaluating the previously discarded SV callers. We found that Sniffles2 worked particularly well for characterizing *RFC1* repeat expansions. Therefore, this tool was added to the pipeline specifically for this purpose.

While several components of the pipeline such as Clair3, CNVpytor, and NanoVar were able to identify variants in some genes with highly homologous pseudogenes, they were not successful in certain genes such as *PMS2 or STRC*. As such, particular components of the Paraphase package were assessed to determine the functionality of this tool in parsing out haplotypes for genes like *PMS2 and STRC*. Since Paraphrase successfully distinguished between variants in highly homologous regions of genes such as *PMS2* from pseudogenes such as *PMS2CL*, this tool was included in our pipeline with the intention of employing it only for particular loci such as *PMS2 and STRC* where other pipeline components were unsuccessful.

### Spectrum of known genomic alterations in clinical samples

**FIGURE 3:**
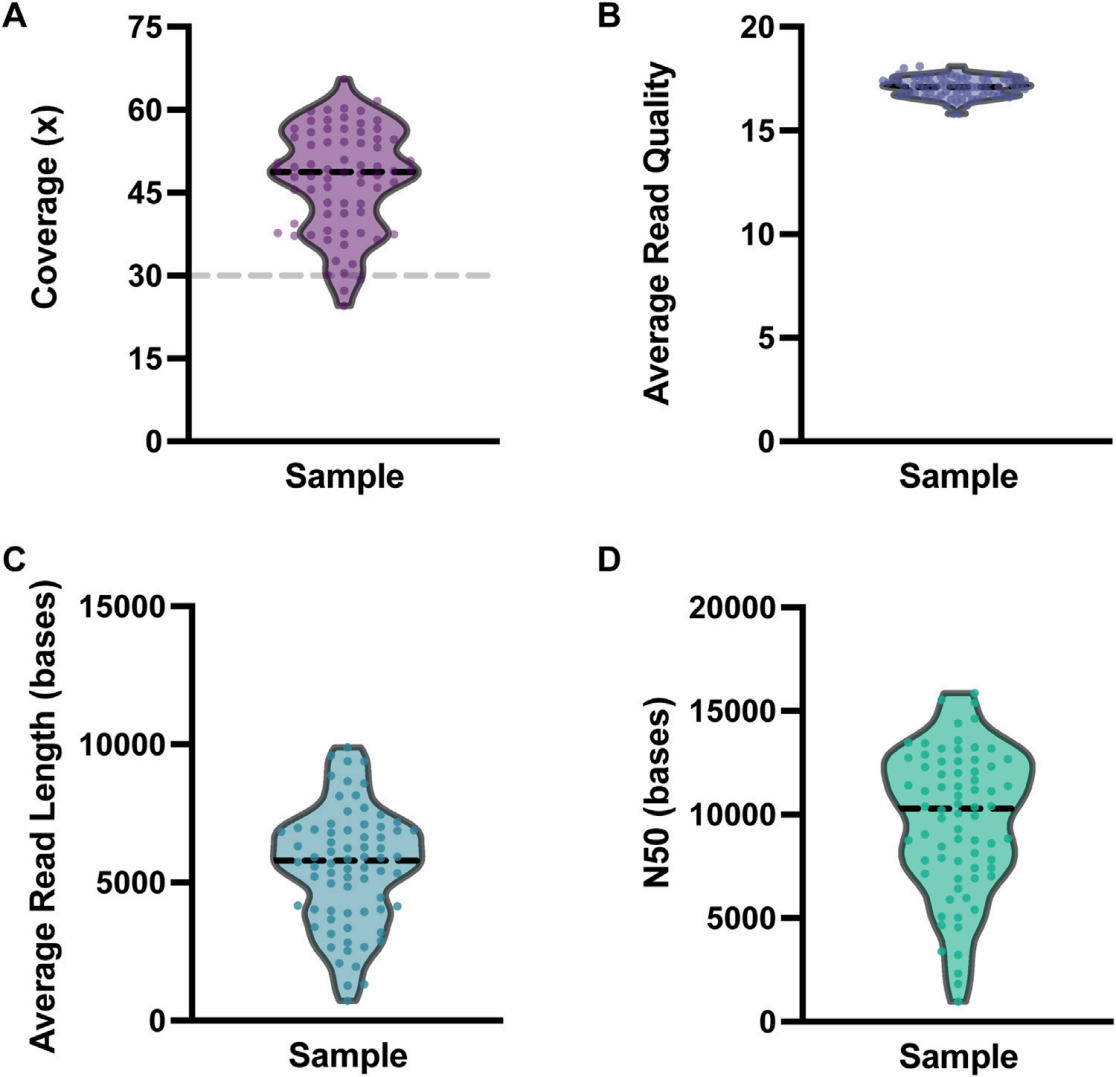
Nanopore sequencing metrics. Violin plots depicting the variability in **(A)** coverage, **(B)** average read quality, **(C)** average read length, and **(D)** N50 for the 72 clinical samples sequenced. For each plot, each dot represents one clinical sample. The dashed black horizontal lines represent the median value for each metric. The dashed gray line on the coverage plot shows the *a priori* target minimum coverage value (30x) for samples in this study.

**FIGURE 4:**
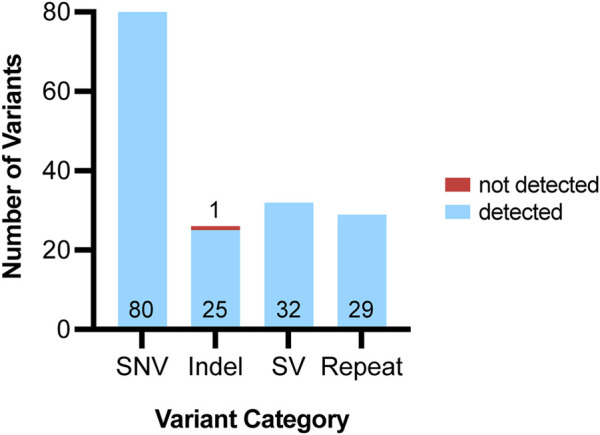
Variants detected using ONT pipeline from 72 clinical samples. Variants assessed using ONT were grouped into four categories: single nucleotide variants (SNV), indels, structural variants (SV) and repeats. For each variant category, the number of variants accurately detected by our pipeline are shown in blue and the variant number is listed at the bottom of each bar. For each variant category, the number of variants that were not accurately detected by our pipeline are shown in red and the variant number is listed above each bar.

All analyzed clinical samples had a genome-wide coverage of approximately 30X; the median N50 was 10,294 bp (Range = 970–15,855 bases). The sequencing metrics are shown in Figure 3. We assessed the performance of long-read sequencing on a cohort of 72 samples from our clinical laboratory. This sample set contained a total of 167 clinically relevant variants that were assessed in this study (Supplementary Table S2; Figure 4). This variant pool consisted of 80 SNVs, 26 indels, 32 SVs, and 29 repeat expansions, which included 14 variants in genes with highly homologous pseudogenes.

### Detection of clinically relevant SNVs

A total of 80 clinically relevant SNVs were assessed by our ONT-based pipeline, all of which were present at a variant allele frequency (VAF) consistent with germline variation (VAF of ∼50% for heterozygous calls and 100% for homozygous calls). All 80 SNVs were accurately identified by Clair3 (Figure 2), indicating that our pipeline has an SNV detection concordance for germline variants of 100% (95% CI: 95%–100%) (Figure 4; Table 1).

### Detection of clinically relevant indels

We assessed 26 clinically significant indels using our ONT-based pipeline (Figure 4). The indel sample set consisted of insertions, deletions, and delins variants ranging from one to 36 bases in size. Clair3 accurately detected 25 of the 26 indels, including seven that fell within homopolymer stretches (4–8 bases in length) and three in genes with highly homologous pseudogenes.

**FIGURE 5:**
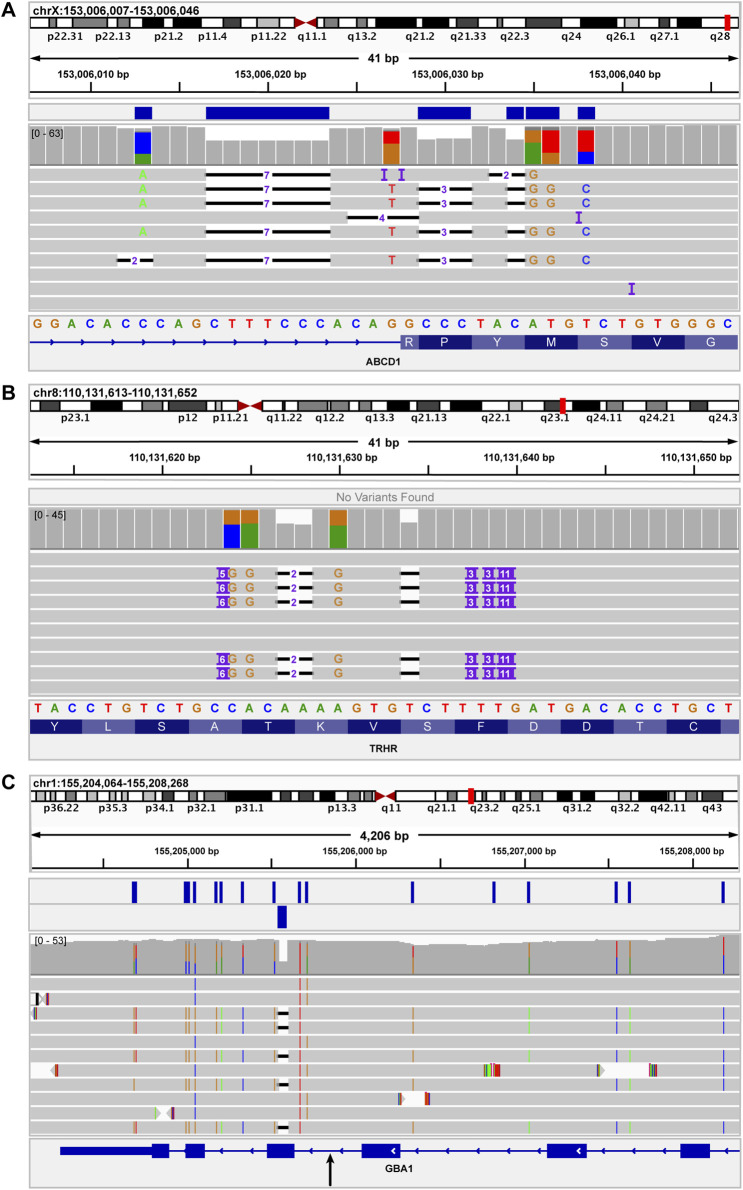
IGV images of indels used to assess functionality of variant callers for indels of different sizes. **(A)** shows the *ABCD1* c.1635-16_1645delinsCACAGACATGTAGGGC variant, which results in a loss of 26 bases and a gain of 16 bases. This variant was accurately detected by the Clair3 component of the pipeline. The blue bars above the coverage data indicate the variants present in the Clair3 VCF. **(B)** shows the *TRHR* c.1137_1152delinsTTTTGTGGCAGGTGCTTGGCTGCCTGCCACAGGCAA variant, which results in a loss of 16 bases and a gain of 36 bases. This was the largest independent indel variant assessed in this study. This indel was not detected by Clair3 nor SV callers. **(C)** shows a complex recombinant allele at the 3′ end of *GBA*. The recombinant allele includes several SNVs as well as a 55-base deletion, representing a gene conversion to pseudogene (*GBAP1*) sequence. The black arrow below the *GBA* sequence at the bottom of the image indicates the approximate junction between reference *GBA* sequence and pseudogene sequence. The genomic region to the right (upstream) of the black arrow is *GBA* sequence. The region to the left (downstream) of the black arrow is the region of gene conversion. The two rows of blue boxes above the coverage data indicate the SNVs called by Clair3 (upper row) and the 55-base deletion called by NanoVar.

Several examples of specific indels assessed using our pipeline are shown in Figure 5. Figure 5A depicts a complex indel in *ABCD1* that was detected and accurately resolved by our pipeline. The only indel variant that was not detected by our pipeline was a complex indel in *TRHR* (Figure 5B). This variant, c.1137_1152delinsTTTTGTGGCAGGTGCTTGGCTGCCTGCCACAGGCAA, includes a loss of 16 bases and a gain of 36 bases. This *TRHR* variant was the largest independent indel variant assessed in this study and it was not called by Clair3. The next-largest indel variant assessed was *BRCA1* c.2472_2490delinsGTAT, which resulted in a loss of 19 bases and a gain of four bases and was accurately called by Clair3.

Figure 5C shows a complex structural variant in *GBA* that contains a 55-base deletion. This *GBA* deletion is part of a complex recombinant allele that also includes several SNVs. As such, the recombinant allele was considered a SV in this study and was not included as an independent indel variant for data analysis. Notably, while Clair3 did not detect the 55-base *GBA* deletion, it was accurately detected by the breakpoint-based caller, NanoVar.

Since NanoVar accurately detected the 55-base deletion component of the *GBA* recombinant allele and Clair3 accurately detected a 19-base indel in *BRCA1*, but all pipeline components failed to detect the 36-base *TRHR* indel, a potential limitation of the current pipeline may be detection of variants between 20 and 55 bases in size, based on this dataset. Given the missed indel call in *TRHR*, the overall indel detection concordance of our pipeline was 96.15% (95% CI: 81%–99%) (Table 1).

### Detection of clinically relevant SVs

Thirty-two clinically relevant SVs were assessed by our pipeline. A combination of breakpoint-based callers (NanoVar and DeBreak) and read depth-based callers (QDNAseq and CNVpytor) was required to accurately call and detect SVs, including copy number variants (CNVs). The combined output from the four callers for SVs and CNVs resulted in an average of about 56,000 raw calls per patient. This number does not indicate unique calls, but rather the sum of all calls across the four callers, including low quality calls that are subsequently filtered out. This combined total also does not account for overlapping calls made by multiple variant calling tools. After implementing a structured filtering strategy, the average number of relevant calls per sample requiring further evaluation and manual review was reduced to about 62 (Figure 1). All 32 SVs were successfully identified by our pipeline, indicating an SV detection concordance of 100% (95% CI: 89%–100%) (Figure 4; Table 1).

### Detection of clinically relevant repeat expansions

**FIGURE 6:**
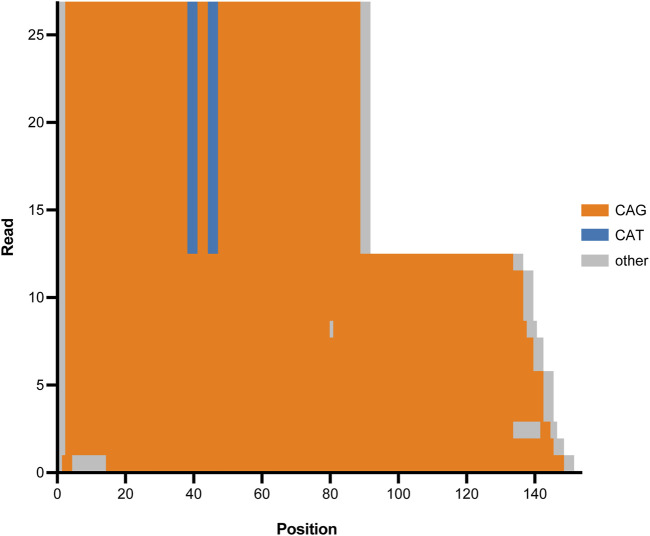
Tandem Genotypes waterfall plot depicting CAG expansion in ATXN1 (SCA1) for one proband. The *x*-axis represents genomic position beginning at the start of the CAG repeat tract in *ATXN1*. Genomic reads are stacked and the y-axis depicts read number. Orange regions represent CAG repeat sequence, blue regions represent CAT interruptions, and grey regions represent genetic sequence that is not CAT nor CAG. Half of the reads (upper region of the graph) show a wild type *ATXN1* allele with two CAT interruptions. The remaining reads (lower region of the graph) show an expanded *ATXN1* allele that does not have protective CAT interruptions and has expanded into the pathogenic range causative of spinocerebellar ataxia type 1 (SCA1).

The validation cohort included 29 clinically significant repeat expansion variants. Our pipeline accurately detected and resolved all 29 repeat expansions, demonstrating a detection concordance of 100% (95% CI: 88%–100%) (Table 1). Tandem Genotypes software correctly identified clinically significant repeat expansions in *ATXN1, ATXN3, ATXN7, DMPK, FGF14, FMR1, FXN*, and *HTT*. Figure 6 depicts the wild type and expanded pathogenic CAG repeat sequences detected in a case of autosomal dominant Spinocerebellar ataxia type 1. All genes with expanded repeats in protein coding regions were accurately detected and sized. Tandem Genotypes also detected larger non-coding expansions, including *FXN* expansions greater than 3,000 nucleotides in length.

Included in these 29 pathogenic repeats were six *RFC1* expansions, all of which were accurately detected by Sniffles2 but were missed by Tandem Genotypes. Importantly, manual review also showed that the expansion had converted from wild type sequence AAAAG to pathogenic sequence AAGGG.

### Detection of clinically relevant variants in genes with highly homologous pseudogenes

**FIGURE 7:**
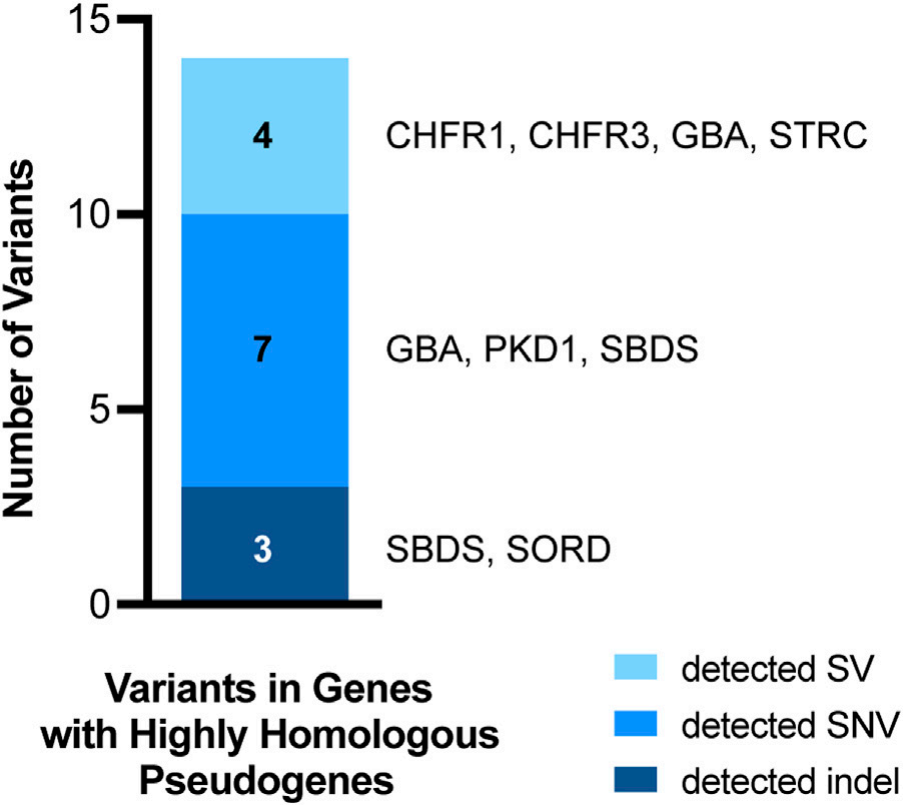
Variants in genes with highly homologous pseudogenes. Fourteen of the 167 variants from clinical samples assessed in this study occurred in genes with highly homologous pseudogenes. All 14 of these variants were detected by our pipeline. The variant types are coded by color and the specific genes in which the variants occurred are listed to the right of each bar segment.

Among the clinical samples assessed, there were 14 clinically significant variants in genes with highly homologous pseudogenes (Figure 7). These variants included SNVs, SVs, and indels in *STRC, CHFR1, CHFR3, SBDS, GBA, PKD1, and SORD*. Importantly, several of these variants could not be detected by short-read NGS and required specialized supplementary assays. The ten SNV and indel variants in genes with highly homologous pseudogenes were detected accurately by the Clair3 component of the ONT-based pipeline. The four SVs were called by Clair3, CNVpytor, and/or Paraphase (Supplementary Table S2). As such, our pipeline was able to detect all these variants in genes with highly homologous pseudogenes, including those that required supplemental methods previously, resulting in a detection concordance of 100% (95% CI: 78%–100%) (Table 1). Thus, detection of variants in genes with highly homologous pseudogenes is a notable strength of the ONT-based pipeline when compared to short-read NGS.

Since our laboratory routinely offers sequence analysis of *PMS2* by long-range PCR (LR-PCR), we focused specifically on our pipeline’s ability to differentiate between variants in *PMS2* and its pseudogene, *PMS2CL*. Paraphase (Chen X. et al., 2023) (PMID: 36669496) is a computational tool specifically designed for genotyping a small set of genes with homologous pseudogenes, including *PMS2*. LR-PCR results were used as a validation tool for providing orthogonal confirmation of results when possible. A key advantage of Paraphase is its ability to infer distinct *PMS2* and *PMS2CL* haplotypes. Five of the 72 samples had orthogonal LR-PCR data available for *PMS2* comparison with ONT outputs. Therefore, we elected to perform a concordance assessment specifically for Paraphase calls with a VAF > 0.2 and a quality score >1. In four of the five samples, all calls that satisfied these criteria were concordant between the nanopore and LR-PCR outputs. Of note, the concordance for other calls, such as those within non-coding homopolymer stretches greater than 10 bases long was lower. Typically, variants within these deep intronic non-coding homopolymer stretches are not expected to be clinically relevant.

Importantly, in one of the five samples, Paraphase identified an intronic block of 18 variants that were not called by LR-PCR. Notably, this is a region of the gene where variants are not analyzed and reported clinically. Paraphase accurately established these calls as being present in *PMS2* despite having substantial overlap with *PMS2CL*. Since nanopore sequencing and Paraphase allow for reads to be “laddered” together to infer distinct haplotypes, this tool allowed us to conclude that the calls made by Paraphase but not LR-PCR represented a true gene conversion of *PMS2* such that it contains a large but clinically insignificant region of *PMS2CL*.

### Cases resolved using ONT

In four cases where short-read NGS findings were not sufficient for a molecular diagnosis in the proband, our ONT-based pipeline was able to accurately detect and resolve variants leading to a complete clinical diagnosis.

**FIGURE 8:**
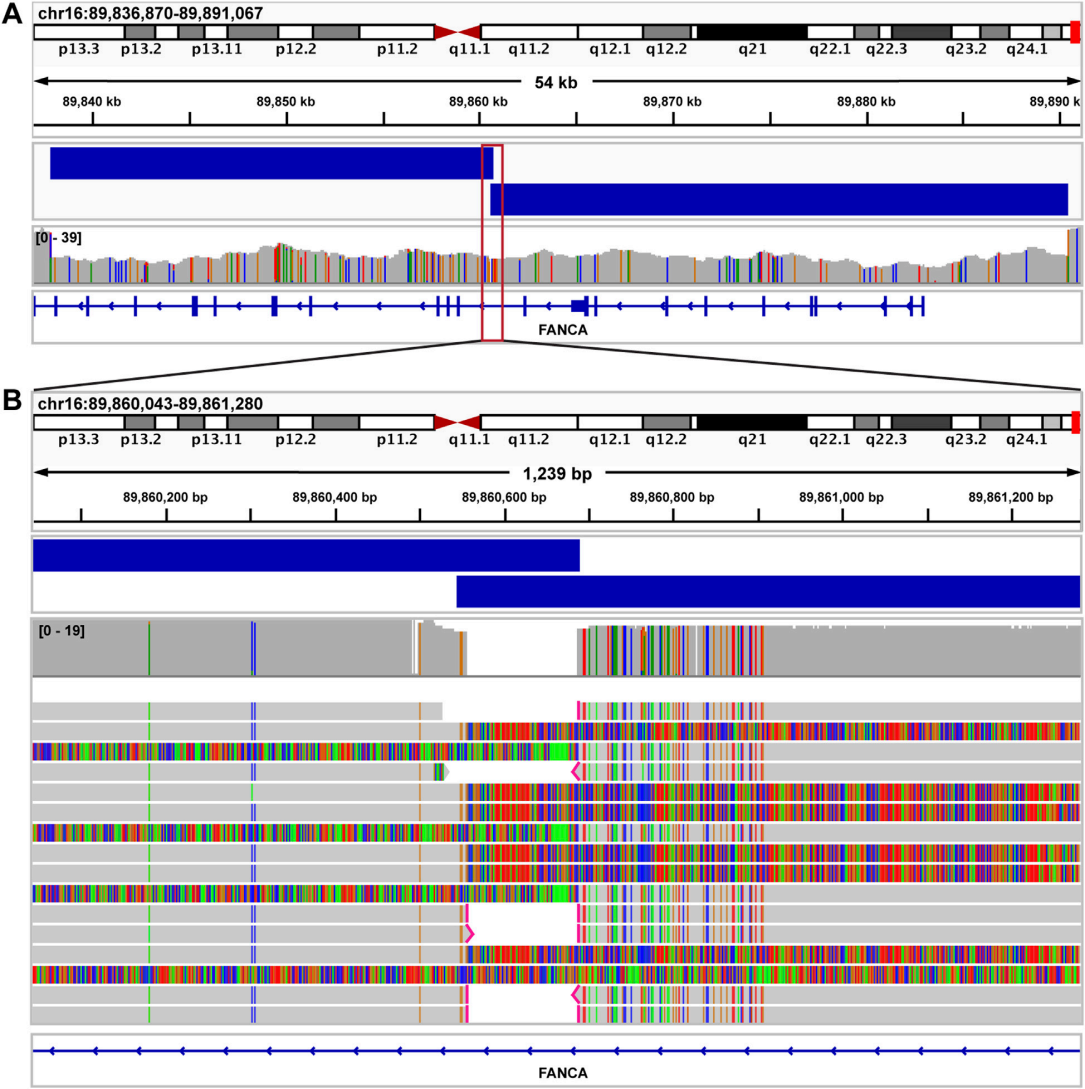
Fanconi anemia case resolved using long read sequencing. **(A)** IGV image showing exons 1–26 of *FANCA* in a sample from a patient with a clinical diagnosis of Fanconi anemia. By short read NGS, a single deletion call was made spanning exons 1–23. Nanopore sequencing identified two distinct *FANCA* deletions in trans (exons 1–11 and exons 12–23), with a 140 bp overlap (blue bars above coverage data). Both deletions were called accurately by Debreak. The red box depicts the genomic region magnified in Figure 7B. **(B)** IGV image showing the region surrounding the 140 bp overlap of both *FANCA* deletions.

In the first case, our current short-read NGS clinical pipeline identified what appeared to be a single heterozygous *FANCA* deletion (exons 1–23), in a 6-year-old male with a clinical presentation consistent with Fanconi anemia. A second pathogenic variant in *FANCA* was not detected. In contrast, our ONT-based sequencing pipeline detected two distinct *FANCA* deletions in trans (exons 1–11 and exons 12–23), with a 140 base pair overlap (Figures 8A,B), allowing for a complete molecular diagnosis for the proband.

The second case is a 62-year-old-male with neurologic symptoms consistent with ataxia. Prior testing via a short tandem repeat panel was negative for this patient, albeit before the discovery of Spinocerebellar ataxia 27B (SCA27B) (Pellerin et al., 2023; Rafehi et al., 2023) (PMID: 36516086, 37267898). ONT-based testing detected a pathogenic GAA repeat expansion in *FGF14*, indicating a molecular diagnosis of SCA27B for this proband. Subsequently, this patient received *FGF14* short tandem repeat testing at an external laboratory via his clinical care team and the results were consistent with our finding.

**FIGURE 9:**
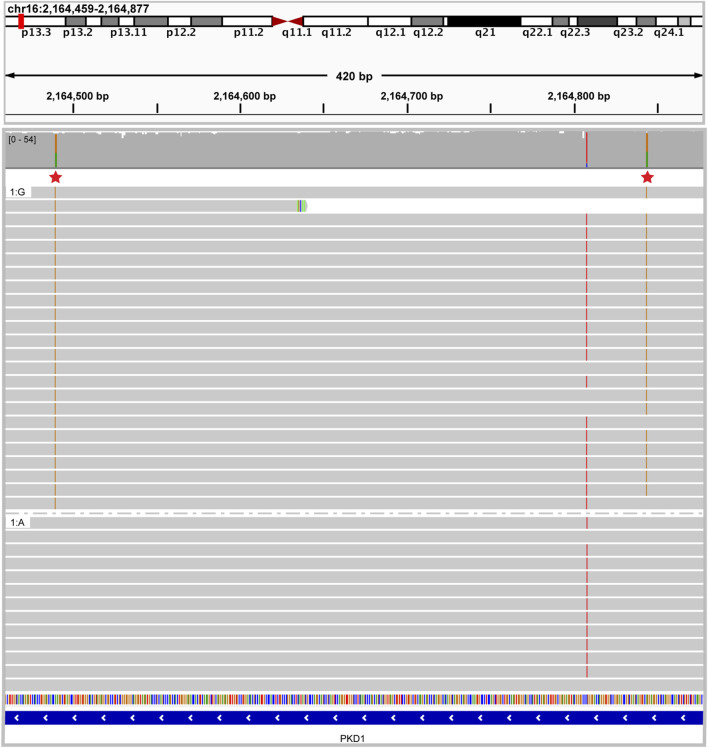
Caroli syndrome case resolved using long read sequencing IGV image showing two heterozygous pathogenic *PKD1* variants in cis. Reads are grouped by nucleotide at chr16:2164490 demonstrating that all the reads with one pathogenic variant share the other pathogenic variant.

In the third case, panel-based NGS testing of *PKD1, PKD2* and *PKHD1* performed in a 12-year-old female with Caroli’s syndrome identified two heterozygous pathogenic variants in the *PKD1* gene. It was unclear whether these variants were present in cis or in trans. ONT-based sequencing clearly showed that these variants were present in cis as the result of a gene conversion event that incorporated pseudogene sequence into the *PKD1* gene (Figure 9). It has been shown that the presence of two *PKD1* variants in trans is associated with more severe disease and poorer prognosis (Ali et al., 2015) (PMID: 25880449). Therefore, establishing the cis phase of the variants in this proband was vital for informed clinical management and provided a more accurate estimate of disease progression.

**FIGURE 10:**
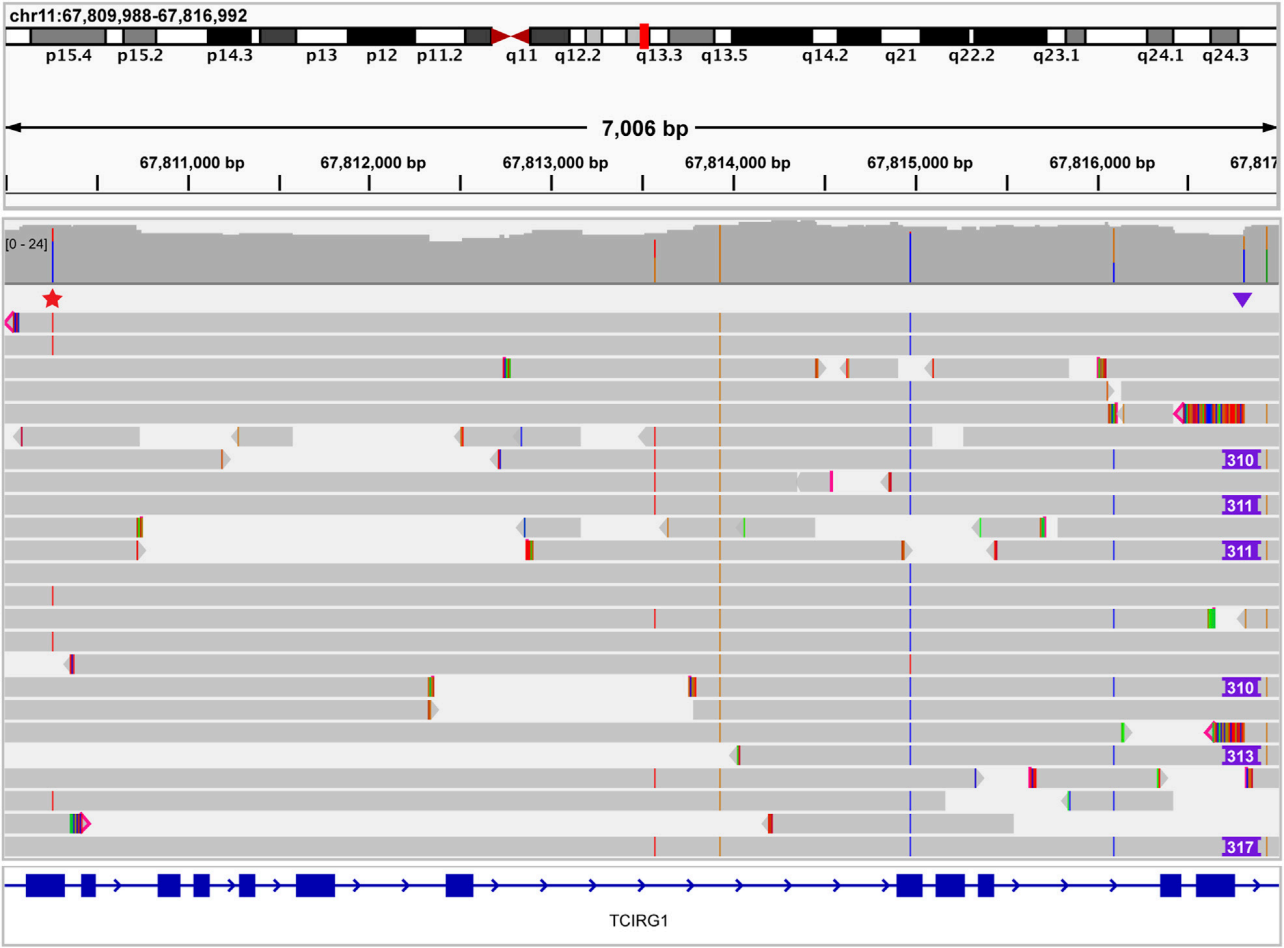
Osteopetrosis case resolved using long read sequencing. IGV image showing a single heterozygous pathogenic nonsense *TCIRG1* variant (red star above reads on left side of image) that was identified previously by an external laboratory. Long read sequencing identified a *TCIRG1-*disrupting Alu insertion (purple triangle above reads on right side of image) in trans, leading to a definitive molecular diagnosis. No spanning reads have both the nonsense variant and the Alu insertion, confirming the trans conformation of these variants.

To further demonstrate the prospective clinical utility of this technology, we analyzed a single case that had not been definitively resolved with prior short-read sequencing. The proband was a one-year-old male with a clinical diagnosis of osteopetrosis. Prior to referral to our institution, the patient had short-read whole genome sequencing performed at an external laboratory. This testing identified a single heterozygous c.346C>T (p.Gln116*) nonsense variant in the *TCIRG1* gene. A second clinically significant *TCIRG1* variant was not identified by the external testing. The patient was referred to our institution for potential bone marrow transplantation based on a presumed diagnosis of autosomal recessive *TCIRG1*-related osteopetrosis. Upon receiving a new sample from the proband and both parents, targeted testing of the parental samples revealed that the c.346C>T (p.Gln116*) variant was paternally inherited. Short-read whole genome sequencing performed at MDL provided evidence of a maternally inherited potential structural variant, but the exact nature of the variant could not be resolved. This sample was sequenced with ONT and a novel Alu insertion was identified at chr11:67816810 (hg19). This Alu insertion was located 49 bases from the exon 15/intron 15 splice boundary. Both breakpoints of this novel Alu insertion were confirmed by Sanger sequencing. The finding of a novel Alu insertion in trans with a pathogenic nonsense variant allowed for a highly probable molecular diagnosis in this case (Figure 10). This insertion was classified as a variant of uncertain clinical significance by strict application of the ACMG criteria (Richards et al., 2015) (PMID: 25741868). This Alu insertion is an example of a variant that is difficult to fit into an interpretive framework that is very specifically designed for sequence variants and small indels. However, in consensus conversations with the clinical providers caring for this patient, we strongly believe that this variant represents a diagnostic finding, which was reflected on the final report. Overall, we chose a conservative formal interpretation and classification given the lack of functional evidence regarding this novel intronic insertion. Of note, because this Alu insertion variant could not be formally classified as clinically significant, this case was not included in the table of clinically significant variants.

## Discussion

This is one of the largest clinical validations performed using long-read sequencing technology to date. Importantly, we successfully combined eight variant callers and developed a pipeline capable of comprehensively detecting a wide range of genetic variants, which is essential for clinical diagnosis of inherited disorders. We also successfully adapted and optimized short-read NGS callers like CNVpytor for a long-read pipeline and have demonstrated that integrating multiple variant callers is essential for utilizing long-read sequencing in clinical diagnostics to detect various genetic alterations. In addition to detecting different types of variants, we have demonstrated that long read sequencing can identify and resolve several complex variants that were not detected by conventional methods, including short read NGS.

Despite the slightly lower analytical sensitivity for small sequence variants seen with long-read sequencing of GIAB sample NA12878 as compared to short-read sequencing, currently there is no single genotyping platform that can match the overall performance of our ONT-based pipeline in the detection of a wide array of genomic alterations. On closer analysis of the 0.5% lower analytical sensitivity for the NA12878 sample, we found that the missed calls did not appear to cluster in any specific gene or region. The missed calls were mostly due to skewed VAFs that were outside the range typically associated with heterozygous calls (Supplementary Figure S1). Notably, multiple iterations of adjusting Clair3 settings were unable to improve detection of these variants. These skewed VAFs were predominantly in the coding regions of the genes and not impacted by homopolymer regions. We recognize that restricting our analysis to coding exons may have reduced the number of artifacts related to homopolymer elements, as the majority of homopolymer elements reside in non-coding regions of the genome. Many of the indel discrepancies did occur adjacent to homopolymer elements, but for SNVs in coding exons, there was no relation to homopolymer content. Due to homopolymer stretches being overrepresented in non-coding sequence, we would expect the analytical sensitivity and specificity to be slightly reduced if non-coding regions were included.

With respect to our clinical cohort of 72 samples, 100% of the clinically relevant small indels (<20 bases) and SNVs were identified by our ONT-based pipeline. Notably, this cohort included a sample with a CFTR intron 9 homopolymer 5T allele, which was used to challenge the pipeline performance and this variant was accurately detected.

There is also an opportunity for continued chemistry and bioinformatics improvement of our pipeline as we move towards larger-scale clinical implementation. While significant advancements have been made in the ONT sequencing chemistry and hardware, we recognize that frequently changing tools can present a challenge for clinical diagnostic laboratories. We acknowledge that long read sequencing chemistries and available software to analyze sequencing data have been rapidly improving and anticipate that it will continue to evolve in the future. While re-analyzing the samples in this project with the latest available versions of the pipeline’s bioinformatics components was not feasible within the scope of this project, we plan to continually assess our ONT pipeline components for optimal clinical performance as we do for the laboratory’s other clinical genomics test offerings.

We will assess future versions of sequencing chemistries and software as they become available, evaluate their impact on variant calling accuracy and make periodic updates to our clinical pipeline as indicated.

### SNVs

All 80 SNVs evaluated in clinical samples were accurately detected, thus showing a 100% detection concordance for clinically relevant SNVs (Figure 4; Table 1). To stress test the pipeline and assess the ability of our platform to detect variants at non-germline VAFs, we utilized an incidental finding of polycythemia vera in one of the samples studied. The JAK2 c.1849G>T (p.Val617Phe) variant, observed at a VAF of 24% in peripheral blood on prior short-read testing, was not detected by the variant callers in our pipeline. Manual review of the BAM file confirmed that the variant was present in sequencing reads for this sample. The inability of our pipeline to detect this mosaic variant is not surprising, given that the pipeline was optimized for detecting germline mutations. Therefore, reliable detection of constitutional mosaic findings may be limited with the current ONT-based pipeline.

### Indels and SVs

In several cases with complex SVs, although a diagnostic finding had been reported based on short-read NGS or other techniques, a full characterization of these complex variants could not be completed using existing clinical methods. In these cases, our ONT-based pipeline helped us to clarify the exact genes involved in a particular SV and better understand the ways these complex SVs altered cellular biology and contributed to proband phenotypes. These SVs were detected using a combination of Clair3, breakpoint-based callers, and read-depth-based callers. We were able to develop an effective SV call filtering strategy that eliminated a majority of the superfluous calls, allowing us to focus on reviewing those with the highest likelihood of being clinically relevant, thus allowing for a high detection concordance of clinically relevant SVs (Figure 1). This filtering strategy is critical for establishing a workflow that can be practically implemented in clinical laboratories. A combination of both read-depth and breakpoint-based callers was used; Clair3 is our default genotyper, while the primary SV callers are NanoVar and DeBreak. DeBreak could accurately detect SVs between 100 kb and 361 kb, but large deletions and duplications (>750 kb) mediated by repetitive elements and those extending to the chromosome centromere or telomere could not be routinely detected by this caller. NanoVar calls were only used to bridge the gap between the maximum for Clair3 (50 bases) and the minimum for DeBreak (100 bases). In contrast, the general purpose SV caller, Sniffles2, showed poor overall performance in detection of complex SVs (Supplementary Figure S3) and was therefore not included as a primary SV caller in our pipeline. It is unclear as to exactly why Sniffles2 did not perform as well in detecting SV breakpoints. This could be due to the use of complex multi-component SVs intended to challenge the limitations of the SV-calling tools as our pilot dataset. Our data therefore suggests that while Sniffles2 may work well for detecting simple deletions and duplications, it showed less than optimal performance for detection of the complex SVs included in our dataset.

As the first step in our SV filtering strategy, we restricted each of the caller’s outputs to a subset of calls related to its variant calling strengths. Our second filtering step entailed identification of variants that were most likely to have an effect on Mendelian disease; another filtering step was then performed to restrict the calls to genes that have a human phenotype (Supplementary Table S1). A final set of logic rules was then applied for accurate detection of a pathogenic SV. This list is not patient- or sample-specific; therefore, this strategy can be adapted for any clinical scenario. We recognize that there are unique complex structural variants, so we maintain all raw outputs for possible manual review in cases with high levels of clinical suspicion.

A 55-base deletion, which was part of the complex SV in *GBA* was not detected by Clair3; but was detected by the breakpoint-based caller NanoVar (Figure 5C). However, the 36-base *TRHR* indel (Figure 5B) was missed both by Clair3 and NanoVar. Manual review of the BAM file showed that this *TRHR* indel was present, indicating that the miss was not due to an error in sequencing. Unfortunately, there were no other SVs in the clinical dataset between 20 and 55 base pairs in length that could be assessed to further narrow the size window of variants this pipeline has difficulty detecting. Thus, reduced capacity to detect indels between 20 and 55 bp may be a potential limitation of our current bioinformatics pipeline that will need to be addressed in future optimization of this clinical workflow.

### Repeat expansions

For analysis of repeat expansions, one specific challenge is that the mere presence of an expanded repeat does not always correlate with pathogenicity. In addition to size of the repeat expansion, nucleotide content is often critical for predicting disease penetrance, severity, age of onset in affected individuals as well as meiotic instability. Using CANVAS as an example we demonstrated that our pipeline was not only able to detect clinically relevant expanded repeats in *RFC1*, but also provided nuanced information about the content. Two key components are required to confer molecular pathogenicity in CANVAS: the repeat content must change from an AAAAG repeat sequence to one of several pathogenic pentanucleotide repeat sequences and the repeat length must expand from a nonpathogenic size (typically 11–200 repeats) to greater than 400 repeats (Delforge et al., 2024) (PMID: 38627134). Indeed, manual review of the BAM files from samples with known pathogenic *RFC1* expansions showed that the reads containing the pathogenic repeats had altered from wild type pentanucleotide sequence AAAAG, to pathogenic pentanucleotide repeat sequence, AAGGG, indicating that the genotyping pipeline was able to accurately measure the size as well as content of this region for clinical diagnostic purposes. Importantly, we demonstrate that Tandem Genotypes was able to identify all the tested repeat expansions in our cohort but was unable to accurately detect all the pathogenic *RFC1* repeats tested. Expanded *RFC1* repeats, implicated in CANVAS, were accurately detected only by Sniffles2. Interestingly, This demonstrates the need to incorporate multiple different callers into a clinical pipeline for detection of clinically relevant variants to achieve high confidence in detection of clinically relevant repeat expansions.

### Genes with highly homologous pseudogenes

As indicated in Figure 7, our pipeline was able to detect all variants in genes with highly homologous pseudogenes. The majority of these variants were detected by Clair3, Nanovar, and CNVpytor. The Paraphase tool was used to assist with variant detection in a specific subset of genes requiring a haplotype based approach, such as *PMS2* and *STRC*, which is in alignment with the UMN MDL’s currently available test offerings. When the laboratory eventually transitions to using a newer reference genome (discussed below), the available Paraphase loci offerings supported for that reference will be evaluated for potential clinical implementation.

## Conclusion and future directions

Despite previously being considered suboptimal for clinical applications due to relatively low base calling accuracy, recent improvements in the ONT technology along with decreasing costs and shorter turnaround time have made it an attractive tool for several genomics applications. ONT sequencing is being increasingly used in clinical microbiology laboratories, particularly in the study of infectious diseases, detection of drug resistance, identification of rare and unknown pathogens, as well as real-time genomic surveillance (Marx, 2023; Park et al., 2021) (PMID: 36635542, 34211026). However, its use as a diagnostic clinical test for human genetic disorders has been limited. While it has been utilized to complement the findings of short-read NGS and provide additional orthogonal confirmation (Kaplun et al., 2023) (PMID: 37152986), long-read sequencing has not been used as a standalone method for comprehensive clinical genetic testing. We have developed a comprehensive workflow that is optimized for detection of several different types of genomic variants. Hence, this study is the first of its kind wherein we use ONT-based long-read sequencing as a single comprehensive testing platform for WGS-based clinical testing.

A key highlight of our custom pipeline was the use of eight different variant callers; while certain SV calls were made by more than one caller, all components were essential to achieve high concordance with other reference methods for detection of clinically relevant variants. However, the use of eight callers resulted in multiple output formats, in this case VCFs generated by various callers. Future work will focus on restructuring and formatting the individual outputs for merging into a single VCF that can be used for variant interpretation. This will enable a streamlined workflow capable of identifying a wide spectrum of genetic variation that can be scaled to analyze the vast majority of clinical samples sent for molecular diagnostics. Our clinical workflow will also incorporate manual review of specific complex loci (such as *RFC1*) to optimize its clinical utility.

Our laboratory is also considering the use of newer human reference builds, GRCh38 or Telomere-to-Telomere (T2T), which provide a more thorough and accurate characterization of the human genome along with improved representation of structural variants and complex genomic regions (Guo et al., 2017; Nurk et al., 2022; Aganezov et al., 2022) (PMID: 28131802, 35357919, 35357935). At present, all clinical analyses and reporting infrastructure in our laboratory use GRCh37/hg19 as human reference. As one of the main goals of this project is to make this pipeline available for immediate clinical use, GRCh37/hg19 will remain our human reference sequence to maintain internal consistency and to ensure that our results can be accurately compared to our current clinical pipelines. However, we plan to take the necessary steps to transition this pipeline and our other current clinical pipelines to GRCh38 or T2T in the near future.

With respect to economic efficiency, the current approximate cost of ONT sequencing and producing the raw sequencing data is between $800 and $1,200. This does not include the costs for bioinformatic analysis, professional interpretation, and data storage. This analysis is more cost effective than our current short-read whole genome sequencing platform because it requires fewer orthogonal assays such as repeat expansion analysis to rule out differential diagnoses than our short-read platform. Furthermore, when the assay is clinically implemented, we anticipate that the associated larger batch sizes will result in lower costs per sample.

When it comes to the diagnosis of certain rare diseases, the genetic assessment process can be complex and intricate. A classic example would be hereditary cerebellar ataxia, which can have many putative causal genes, including a constantly growing list of repeat expansion disorders. The diagnostic yield in these patients, even after the advent of short-read NGS, is low, and there continues to be a diagnostic gap (Cortese et al., 2019; Tenorio et al., 2024) (PMID: 30926972, 37950147). To improve the diagnostic yield in such rare diseases, it will be essential to develop a one-stop comprehensive genetic test or an assay that can be easily adapted to the identification of new repeat expansion disorders. Our work has shown that long-read sequencing can be leveraged to address this unmet need.

We demonstrate the feasibility of a single cost-effective long-read sequencing platform for the diagnosis of rare genetic disorders caused by a broad spectrum of genetic variation. Offering this comprehensive testing clinically will improve turnaround time and enhance patient care by limiting the number of separate tests required for diagnosis and would allow for future review of the raw data for additional diagnostic testing, should new clinical indications arise. Our validation study has shown that ONT sequencing can efficiently detect a wide range of reportable mutations, thus ensuring rapid turnaround for whole genome sequencing-based clinical genetic testing.

## Data Availability

The bioinformatics code is available in a publicly accessible repository. This data can be found here: DOI: 10.5281/zenodo.14532101, https://zenodo.org/records/14805778. All other sequencing data is available in the main and supplementary sections of the manuscript.

## Supplementary material

SUPPLEMENTARY FIGURE S1 Representative ‘missed’ ONT SNV calls. IGV image showing two SNVs in coding regions for GIAB sample NA12878. The upper set of read data for each variant is from ONT sequencing and the lower set of read data is from short-read sequencing. These SNVs were not accurately called by our ONT pipeline, but they were accurately detected by our short-read pipeline. Both SNVs shown are representative of the skewed VAFs that caused variants called on the short-read pipeline to be missed by the ONT pipeline. These skewed VAFs occurred predominantly in coding regions and were not impacted by homopolymer stretches.

## References

[B1] Aganezov S. Yan S. M. Soto D. C. Kirsche M. Zarate S. Avdeyev P. (2022). A complete reference genome improves analysis of human genetic variation. Science 376, eabl3533. 10.1126/science.abl3533 35357935 PMC9336181

[B2] Ali H. Hussain N. Naim M. Zayed M. Al-Mulla F. Kehinde E. O. (2015). A novel PKD1 variant demonstrates a disease-modifying role in trans with a truncating PKD1 mutation in patients with autosomal dominant polycystic kidney disease. BMC Nephrol. 16, 26. 10.1186/s12882-015-0015-7 25880449 PMC4357204

[B3] Chen X. Harting J. Farrow E. Thiffault I. Kasperaviciute D. Genomics England Research C. (2023). Comprehensive SMN1 and SMN2 profiling for spinal muscular atrophy analysis using long-read PacBio HiFi sequencing. Am. J. Hum. Genet. 110, 240–250. 10.1016/j.ajhg.2023.01.001 36669496 PMC9943720

[B4] Chen Y. Wang A. Y. Barkley C. A. Zhang Y. Zhao X. Gao M. (2023). Deciphering the exact breakpoints of structural variations using long sequencing reads with DeBreak. Nat. Commun. 14, 283. 10.1038/s41467-023-35996-1 36650186 PMC9845341

[B5] Cortese A. Simone R. Sullivan R. Vandrovcova J. Tariq H. Yau W. Y. (2019). Biallelic expansion of an intronic repeat in RFC1 is a common cause of late-onset ataxia. Nat. Genet. 51, 649–658. 10.1038/s41588-019-0372-4 30926972 PMC6709527

[B6] Danecek P. Bonfield J. K. Liddle J. Marshall J. Ohan V. Pollard M. O. (2021). Twelve years of SAMtools and BCFtools gigascience 10. Gigascience 10, giab008. 10.1093/gigascience/giab008 33590861 PMC7931819

[B7] De Coster W. Rademakers R. (2023). NanoPack2: population-scale evaluation of long-read sequencing data. Bioinformatics 39, btad311. 10.1093/bioinformatics/btad311 37171891 PMC10196664

[B8] Delforge V. Tard C. Davion J. B. Dujardin K. Wissocq A. Dhaenens C. M. (2024). RFC1: motifs and phenotypes. Rev. Neurol. (Paris) 180, 393–409. 10.1016/j.neurol.2024.03.006 38627134

[B9] Fernandez-Marmiesse A. Gouveia S. Couce M. L. (2018). NGS technologies as a turning point in rare disease research, diagnosis and treatment. Curr. Med. Chem. 25, 404–432. 10.2174/0929867324666170718101946 28721829 PMC5815091

[B10] Gorzynski J. E. Goenka S. D. Shafin K. Jensen T. D. Fisk D. G. Grove M. E. (2022). Ultrarapid nanopore genome sequencing in a critical care setting. N. Engl. J. Med. 386, 700–702. 10.1056/NEJMc2112090 35020984

[B11] Guo Y. Dai Y. Yu H. Zhao S. Samuels D. C. Shyr Y. (2017). Improvements and impacts of GRCh38 human reference on high throughput sequencing data analysis. Genomics 109, 83–90. 10.1016/j.ygeno.2017.01.005 28131802

[B12] Hartman P. Beckman K. Silverstein K. Yohe S. Schomaker M. Henzler C. (2019). Next generation sequencing for clinical diagnostics: five year experience of an academic laboratory. Mol. Genet. Metab. Rep. 19, 100464. 10.1016/j.ymgmr.2019.100464 30891420 PMC6403447

[B13] Hook P. W. Timp W. (2023). Beyond assembly: the increasing flexibility of single-molecule sequencing technology. Nat. Rev. Genet. 24, 627–641. 10.1038/s41576-023-00600-1 37161088 PMC10169143

[B14] Kaplun L. Krautz-Peterson G. Neerman N. Stanley C. Hussey S. Folwick M. (2023). ONT long-read WGS for variant discovery and orthogonal confirmation of short read WGS derived genetic variants in clinical genetic testing. Front. Genet. 14, 1145285. 10.3389/fgene.2023.1145285 37152986 PMC10160624

[B15] Kielbasa S. M. Wan R. Sato K. Horton P. Frith M. C. (2011). Adaptive seeds tame genomic sequence comparison. Genome Res. 21, 487–493. 10.1101/gr.113985.110 21209072 PMC3044862

[B16] Liu C. Yang X. Duffy B. F. Hoisington-Lopez J. Crosby M. Porche-Sorbet R. (2021). High-resolution HLA typing by long reads from the R10.3 Oxford nanopore flow cells. Hum. Immunol. 82, 288–295. 10.1016/j.humimm.2021.02.005 33612390

[B17] Lu H. Giordano F. Ning Z. (2016). Oxford nanopore MinION sequencing and genome assembly. Genomics Proteomics Bioinformatics 14, 265–279. 10.1016/j.gpb.2016.05.004 27646134 PMC5093776

[B18] Marx V. (2023). Method of the year: long-read sequencing. Nat. Methods 20, 6–11. 10.1038/s41592-022-01730-w 36635542

[B19] Merker J. D. Wenger A. M. Sneddon T. Grove M. Zappala Z. Fresard L. (2018). Long-read genome sequencing identifies causal structural variation in a Mendelian disease. Genet. Med. 20, 159–163. 10.1038/gim.2017.86 28640241 PMC5741540

[B20] Mitsuhashi S. Frith M. C. Mizuguchi T. Miyatake S. Toyota T. Adachi H. (2019). Tandem-genotypes: robust detection of tandem repeat expansions from long DNA reads. Genome Biol. 20, 58. 10.1186/s13059-019-1667-6 30890163 PMC6425644

[B21] Ni Y. Liu X. Simeneh Z. M. Yang M. Li R. (2023). Benchmarking of Nanopore R10.4 and R9.4.1 flow cells in single-cell whole-genome amplification and whole-genome shotgun sequencing. Comput. Struct. Biotechnol. J. 21, 2352–2364. 10.1016/j.csbj.2023.03.038 37025654 PMC10070092

[B22] Nurk S. Koren S. Rhie A. Rautiainen M. Bzikadze A. V. Mikheenko A. (2022). The complete sequence of a human genome. Science 376, 44–53. 10.1126/science.abj6987 35357919 PMC9186530

[B23] Onsongo G. Baughn L. B. Bower M. Henzler C. Schomaker M. Silverstein K. A. (2016). CNV-RF is a random forest-based copy number variation detection method using next-generation sequencing. J. Mol. Diagn 18, 872–881. 10.1016/j.jmoldx.2016.07.001 27597741

[B24] Park S. Y. Faraci G. Ward P. M. Emerson J. F. Lee H. Y. (2021). High-precision and cost-efficient sequencing for real-time COVID-19 surveillance. Sci. Rep. 11, 13669. 10.1038/s41598-021-93145-4 34211026 PMC8249533

[B25] Pedersen B. S. Quinlan A. R. (2018). Mosdepth: quick coverage calculation for genomes and exomes. Bioinformatics 34, 867–868. 10.1093/bioinformatics/btx699 29096012 PMC6030888

[B26] Pellerin D. Danzi M. C. Wilke C. Renaud M. Fazal S. Dicaire M. J. (2023). Deep intronic FGF14 GAA repeat expansion in late-onset cerebellar ataxia. N. Engl. J. Med. 388, 128–141. 10.1056/NEJMoa2207406 36516086 PMC10042577

[B27] Quinlan A. R. Hall I. M. (2010). BEDTools: a flexible suite of utilities for comparing genomic features. Bioinformatics 26, 841–842. 10.1093/bioinformatics/btq033 20110278 PMC2832824

[B28] Rafehi H. Read J. Szmulewicz D. J. Davies K. C. Snell P. Fearnley L. G. (2023). An intronic GAA repeat expansion in FGF14 causes the autosomal-dominant adult-onset ataxia SCA27B/ATX-FGF14. Am. J. Hum. Genet. 110, 1018. 10.1016/j.ajhg.2023.05.005 37267898 PMC10257192

[B29] Richards S. Aziz N. Bale S. Bick D. Das S. Gastier-Foster J. (2015). Standards and guidelines for the interpretation of sequence variants: a joint consensus recommendation of the American college of medical genetics and genomics and the association for molecular Pathology. Genet. Med. 17, 405–424. 10.1038/gim.2015.30 25741868 PMC4544753

[B30] Rudaks L. I. Yeow D. Ng K. Deveson I. W. Kennerson M. L. Kumar K. R. (2024). An update on the adult-onset hereditary cerebellar ataxias: novel genetic causes and new diagnostic approaches. Cerebellum 23, 2152–2168. 10.1007/s12311-024-01703-z 38760634 PMC11489183

[B31] Sedlazeck F. J. Rescheneder P. Smolka M. Fang H. Nattestad M. von Haeseler A. (2018). Accurate detection of complex structural variations using single-molecule sequencing. Nat. Methods 15, 461–468. 10.1038/s41592-018-0001-7 29713083 PMC5990442

[B32] Singh R. R. (2022). Target enrichment approaches for next-generation sequencing applications in oncology. Diagnostics (Basel) 12, 1539. 10.3390/diagnostics12071539 35885445 PMC9318977

[B33] Srivathsan A. Feng V. Suarez D. Emerson B. Meier R. (2024). ONTbarcoder 2.0: rapid species discovery and identification with real‐time barcoding facilitated by Oxford Nanopore R10.4. Cladistics 40, 192–203. 10.1111/cla.12566 38041646

[B34] Stevanovski I. Chintalaphani S. R. Gamaarachchi H. Ferguson J. M. Pineda S. S. Scriba C. K. (2022). Comprehensive genetic diagnosis of tandem repeat expansion disorders with programmable targeted nanopore sequencing. Sci. Adv. 8, eabm5386. 10.1126/sciadv.abm5386 35245110 PMC8896783

[B35] Suvakov M. Panda A. Diesh C. Holmes I. Abyzov A. (2021). CNVpytor: a tool for copy number variation detection and analysis from read depth and allele imbalance in whole-genome sequencing. Gigascience 10, giab074. 10.1093/gigascience/giab074 34817058 PMC8612020

[B36] Tenorio R. B. Camargo C. H. F. Donis K. C. Almeida C. C. B. Teive H. A. G. (2024). Diagnostic yield of NGS tests for hereditary ataxia: a systematic review. Cerebellum 23, 1552–1565. 10.1007/s12311-023-01629-y 37950147

[B37] Tham C. Y. Tirado-Magallanes R. Goh Y. Fullwood M. J. Koh B. T. H. Wang W. (2020). NanoVar: accurate characterization of patients’ genomic structural variants using low-depth nanopore sequencing. Genome Biol. 21, 56. 10.1186/s13059-020-01968-7 32127024 PMC7055087

[B38] Zavodna M. Bagshaw A. Brauning R. Gemmell N. J. (2014). The accuracy, feasibility and challenges of sequencing short tandem repeats using next-generation sequencing platforms. PLoS One 9, e113862. 10.1371/journal.pone.0113862 25436869 PMC4250034

[B39] Zheng Z. Li S. Su J. Leung A. W. Lam T. W. Luo R. (2022). Symphonizing pileup and full-alignment for deep learning-based long-read variant calling. Nat. Comput. Sci. 2, 797–803. 10.1038/s43588-022-00387-x 38177392

[B40] Zook J. M. Catoe D. McDaniel J. Vang L. Spies N. Sidow A. (2016). Extensive sequencing of seven human genomes to characterize benchmark reference materials. Sci. Data 3, 160025. 10.1038/sdata.2016.25 27271295 PMC4896128

