# Supplementary figures and images for: Validation of a comprehensive long-read sequencing platform for broad clinical genetic diagnosis
Source: Front Genet. 2025 May 2;16:1499456. doi: 10.3389/fgene.2025.1499456 (PMC12082127; doi:10.3389/fgene.2025.1499456)

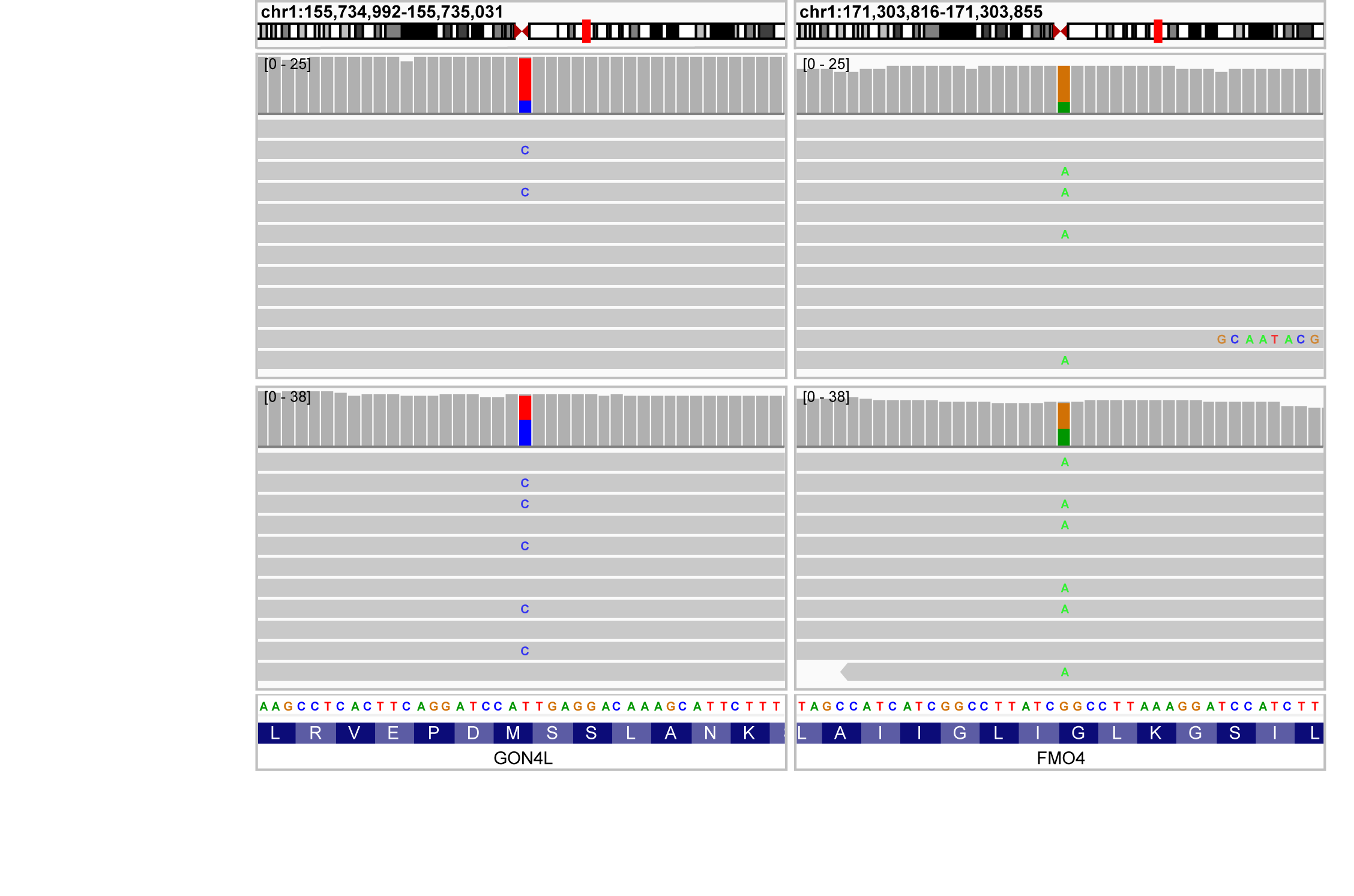

Supplement: Supplementary file 3 [file Image1.tif]
